# Intersections between gender and other relevant social determinants of health inequalities

**DOI:** 10.1080/16549716.2017.1397909

**Published:** 2017-12-23

**Authors:** Isabel Goicolea, Ann Öhman, Carmen Vives-Cases

**Affiliations:** ^a^ Unit of Epidemiology and Global Health, Department of Public Health and Clinical Medicine, Umeå University, Umeå, Sweden; ^b^ Umeå Center for Gender Studies, Umeå University, Umeå, Sweden; ^c^ Department of Community Nursing, Preventive Medicine and Public Health and History of Science, Alicante University, Alicante, Spain; ^d^ CIBER of Epidemiology and Public Health, Barcelona, Spain

The current editorial for the cluster of papers entitled ‘Gender and health inequalities: intersections with other relevant axes of oppression’ includes a final list of twenty accepted papers, concerning gender inequalities in health, and how they interact at different lifetime stages with diverse social determinants of health such as socio-economic status, ethnicity or territory, as well as other intermediate factors such as illness and/or disability. A variety of topics regarding gender inequalities and other social inequalities in health are covered by both qualitative and quantitative empirical research, theoretical papers and review articles. The intersectional approach prevails over other gender theories among published papers in this cluster. In this editorial, we briefly summarize and classify the papers according to their main contributions in this area.

We would like to start by highlighting two papers due to their valuable contribution to **theorizing in intersectionality and health**. First, Hankivsky and co-authors provide an excellent overview of the advances and challenges of intersectionality in health research, offering three concrete examples of how intersectionality has been used to examine complex relationships between biological and social dimensions in the field of health inequality [1]. Second, Wiklund and co-authors draw on intersectional theory as well as on feminist theory on health inequalities and social and cultural variables on health to understand better patients’ perceptions in access to rehabilitation. Of specific value is that they integrate the concept of habitus and cultural capital to the discussion about intersectionality in health research [2].

Among other papers in this cluster, several take an **inter-categorical approach** – i.e. they provisionally adopt existing analytical categories to document relationships of inequality among social groups [3]. Gustafsson’s et al.’s paper ‘Meddling with middle modalities’, for example, analyses inequalities in mental health in Sweden comparing dominant and subordinate middle groups and exploring the importance of intermediate social determinants of health such as health care, material conditions, and violence [4]. Zhang’s paper also takes this approach, to assess health disparities among the elderly in rural China focusing on gender and ethnicity [5]. To a certain extent, Grace´s systematic review analyzing gendered differences in the detection of leprosy [6], and Degerstedt et al.’s analysis of physiotherapeutic interventions and physical activity for children living with cerebral palsy in Northern Sweden could also be included in this group [7].

Other papers in this cluster take an **intra-categorical approach**, using the multiple experiences of oppressed groups to highlight heterogeneity as ways of demonstrating the inadequacy of categories [3]. These papers explore a diversity of experiences; taken together, they tackle several axes of oppression, beyond the traditional ‘gender and race’ dimension [8]. Examples of these papers include Kosia et al.’s study among women living with HIV and AIDS in Tanzania [9], and Dean et al.’s exploration of the sexual and reproductive health rights of disabled women in India [10]. Three papers focus on exploring the lived experiences of women (and men) who are in more vulnerable situations, focusing on gender inequality and violence against women: Kane et al.’s paper on gender relations and reproductive health in Sudan, Madiba’s paper that brings in narratives from married and cohabiting women in rural south Africa, and Mtega et al.’s paper on sex communication among partners in Tanzania [11–13]. Devi Pun’s paper from Nepal explores violence against women, taking the perspective of the community to undercover culturally normalized practices of oppression against women [14].

Especially interesting examples of papers that take an intra-categorical perspective are Logié’s paper exploring the experiences of violence among internally displaced youth in Haiti [15]; and Shannon et al.’s analysis of gender, violence and health in the Amazon of Peru [16]. The first, because it focuses on a category of oppression that has not yet received much attention: displacement. The second, because it takes a historical perspective, bringing colonization and environmental exploitation to the fore in order to understand the experiences of oppression and marginalization. Beyond the division of inter-categorical and intra-categorical, the paper by Guedes et al. constitutes an appreciated example of how an intersectional perspective can illuminate complex problems, such as the intersections of violence against women and violence against children [17].

Helman et al.’s paper on everyday inequalities at home among South African families is an exploration of family dynamics and the reproduction of inequalities. Particularly interesting in this paper is the problematization of categories, especially the category of race and the aim to identify discourses that challenge the status quo [18]. Although this aim for change is in the foundation of intersectionality [19–21], issues concerning resistance, agency, resilience or empowerment are scarce in comparison with papers that tackle different situation of suffering, as was the case in a previous call for papers about gender and health [22]. Among the articles that integrate ‘activism’ or ‘action-orientation’ perspectives, we find Gibbs et al.’s review demonstrating that interventions to prevent HIV and IPV should combine both economic and gender-transformative components [23]; and the paper by Sridharan et al., which highlights the challenges of incorporating gender, equity and rights into the planning processes of international organizations [24]. Of particular relevance for this ‘action-orientation’ perspective are two papers that bring in the voices of activists: Vives-Cases et al. highlight the voices of female representatives of Roma associations to analyze, from their perspective, the different struggles faced by Roma women in relation to violence against women, but also their work against it [25]; and Samuel’s paper concerning the struggle of civic society in strengthening intercultural maternal health in Peru [26].

To conclude, the intersectional theory of gender inequalities in health was brilliantly adopted in the papers forming this cluster. The published papers together contribute to increase knowledge on the complex intersections between different axes of oppression and their relation to health in different populations around the globe. We would like to indicate some future challenges in the research on gender and health and their intersections with other social determinants in health inequalities. As previously mentioned, women are usually present in health research as victims, suffering or at risk of illness and disease. However, women’s agency and increased autonomy in terms of social and economic status is less researched. Therefore, we welcome research for instance on women’s experiences as leaders of social innovations and health advocacy. Further, there remain relevant leading causes of illness that should be further addressed in gender and health research. Other issues of importance are work-related ill health and climate change-related health problems. Also of importance are policy analysis of health prevention and promotion that incorporates a gender theoretical and intersectional lens. Studies that integrate and/or combine other gender and feminist theories, e.g. second-wave feminism, masculinity theories, relational or biosocial approaches would be of interest in a future special issue in Global Health Action.
